# Clinical Efficacy and Learning Curve of Far‐Lateral Approach (FLA) in Uni‐Portal Non‐Coaxial Spinal Endoscopic Surgery (UNSES) in the Treatment of Lumbar Degenerative Diseases: A Prospective Study

**DOI:** 10.1111/os.70395

**Published:** 2026-08-06

**Authors:** Mengchen Yin, Wenlong Yu, Yan Lou, Jianfeng Sha, Yang Xue, Dong Zhang, Kaipeng Huang, Kai Lv, Zhichao Ge, Jianmin Li, Jiajun Wu, Hao Sun, Shuqiang Wang

**Affiliations:** ^1^ Department of Spine, Yueyang Hospital of Integrated Traditional Chinese and Western Medicine Shanghai University of Traditional Chinese Medicine Shanghai China; ^2^ Department of Orthopedics, Changzheng Hospital Second Affiliated Hospital of Naval Medical University Shanghai China

**Keywords:** far‐lateral approach, learning curve, lumbar degenerative diseases, minimally invasive surgery, uniportal non‐coaxial spinal endoscopic surgery

## Abstract

**Background:**

Uniportal non‐coaxial spinal endoscopic surgery (UNSES) via far‐lateral approach (FLA) is an innovative minimally invasive procedure for lumbar degenerative diseases, particularly far‐lateral disc herniation and foraminal stenosis. However, complex lateral lumbar anatomy and strict endoscope‐instrument coordination create a distinct learning curve that may compromise early surgical efficiency and safety. This study aimed to evaluate the efficacy and safety, quantify the learning curve, and to provide clinical guidance for the standardized promotion and application of this technology.

**Methods:**

A total of 40 consecutive patients with lumbar degenerative diseases who underwent UNSES via FLA by a single surgeon between January 2025 and December 2025 were included. All data were analyzed using SPSS 26.0 statistical software (IBM, USA). Primary outcomes included operation time, blood loss, fluoroscopy frequency, and intraoperative complication rate. Secondary outcomes were VAS, ODI, and modified Macnab criteria at 1, 3, and 6 months postoperatively. The learning curve and the inflection point of the learning curve was determined using cumulative sum (CUSUM) analysis. The differences in clinical indicators between early and proficient stage were compared.

**Result:**

Operation time, blood loss, and fluoroscopy times decreased significantly with case accumulation (*p* < 0.05). CUSUM identified an inflection point at the 16th case, after which operation time stabilized at (55.3 ± 8.6) min, much shorter than the early phase (89.5 ± 10.3) min (*p* < 0.001). Before the 16th case, the curve was in an upward trend; after the 16th case, the curve tended to be flat, indicating the proficiency stage. Postoperative VAS and ODI improved significantly than those before surgery at each follow‐up time (*p* < 0.05). There was no significant difference in postoperative VAS score and ODI between the two groups at each follow‐up time point (*p* > 0.05). The total complication rate was 12.5% (5/40), were cured by conservative treatment. The total excellent‐good rate was 90.0% (36/40). L5/S1 and Bertolotti's syndrome were independent factors affecting the learning curve.

**Conclusion:**

UNSES via FLA is a safe and effective minimally invasive technique for treating complex lumbar degenerative diseases. It has a certain learning curve, and the inflection point is about the 16th case. After mastering the key techniques such as anatomical positioning, endoscopic manipulation and hemostasis, the surgeon can gradually reach the proficiency stage, with significantly improved surgical efficiency and clinical efficacy, and controllable complications. This study provides a theoretical basis for the clinical training and technology promotion of UNSES via FLA.

## Graphical abstract

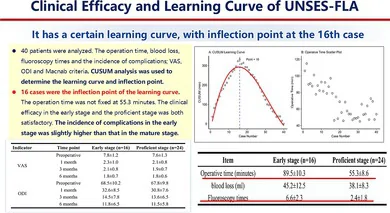

## 1 Introduction

Lumbar degenerative diseases, including lumbar disc herniation, lumbar foraminal stenosis, and far‐out syndrome, are common causes of low back pain and lower extremity radiating pain, which seriously affect the quality of life of patients [1, 2, 3, 4, 5]. In recent years, minimally invasive spinal surgery has gradually replaced traditional open surgery as the preferred treatment due to its advantages of small trauma, less bleeding, rapid recovery, and low complication rate. Uniportal non‐coaxial spinal endoscopic surgery (UNSES) is an innovative minimally invasive spinal surgery technique proposed in recent years, which integrates the advantages of uniportal and non‐coaxial endoscopic technology [6, 7, 8, 9, 10].

The far‐lateral approach (FLA) in UNSES is specially designed for lesions in the foraminal and extraforaminal regions, which can provide a wider surgical field and higher operational flexibility, and is particularly suitable for the treatment of far‐lateral lumbar disc herniation, upper lumbar disc herniation, foraminal stenosis, far‐out syndrome, and Bertolotti's syndrome [11, 12, 13, 14, 15, 16]. Compared with the traditional open far‐lateral approach and single‐axis endoscopic transforaminal approach, the FLA technique has obvious advantages: it adopts a single incision which reduces soft tissue damage; the 30° arthroscope provides a wide field of view, which is conducive to the exposure of the exit nerve root and the removal of the herniated nucleus pulposus; the non‐coaxial design allows independent operation of the endoscope and instruments in the same incision, improving operational flexibility [17]. However, this technique has high requirements for the surgeon's mastery of lumbar lateral anatomy and the coordination ability between endoscopic observation and instrument operation. The complex anatomical structure of the lumbar lateral region, including the nerve root, facet joint, and ligaments, increases the difficulty of the operation. Therefore, the FLA technique has a steeper learning curve than that of open surgery, and the surgical efficiency and safety may be affected in the early stage of learning, which restricts the popularization and application of this technique.

This study prospectively analyzed the clinical data of consecutive patients who underwent FLA technique by a single surgeon, aimed to (i) evaluate the efficacy and safety, (ii) quantify the learning curve, and (iii) determine the inflection point of proficiency of this technology.

## 2 Materials and Methods

### 2.1 Study Design and Participants

**FIGURE 1:**
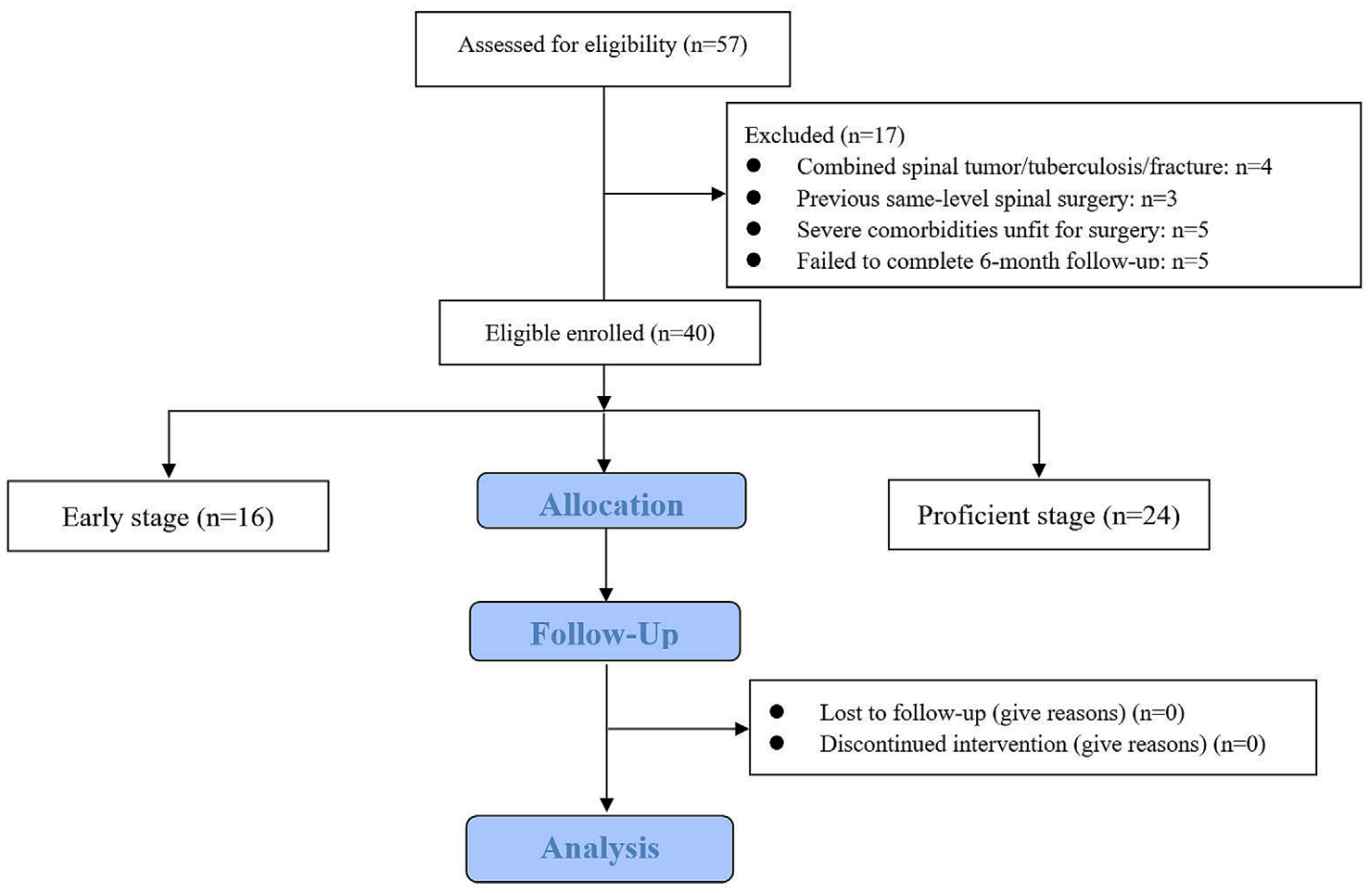
Flow chart of the study.

This was a single‐center, single‐arm, prospective observational study. The study was approved by the Ethics Committee of Yueyang Hospital (YY2024‐65ND). A total of 40 consecutive patients with lumbar degenerative diseases who underwent UNSES via FLA by a single surgeon between January 2025 and December 2025 were included (Figure 1). All participants provided written informed consent before enrollment. The surgeons had more than 10 years of experience in open spine surgery, proficient in the traditional far‐lateral approach, but had no prior experience in UNSES before the first case of this study. Three cadaver specimen practice sessions without live proctor guidance. This clarifies baseline endoscopic naivety and its impact on the steep learning curve. The inclusion criteria were: (1) diagnosed with far‐lateral lumbar disc herniation, upper lumbar disc herniation, foraminal stenosis, far‐out syndrome, and Bertolotti's syndrome; (2) conservative treatment for more than 3 months with poor efficacy; (3) single degenerative lesion without multi‐level severe degeneration requiring combined decompression; and (4) no contraindications to minimally invasive spinal surgery. The exclusion criteria were: (1) combined with other spinal diseases (such as spinal tumor, spinal tuberculosis, or fracture); (2) history of spinal surgery at the same level; (3) severe comorbidities (such as severe heart, lung, or kidney disease) that could not tolerate surgery; and (4) follow‐up time less than 6 months.

### 2.2 Surgical Technique

**FIGURE 2:**
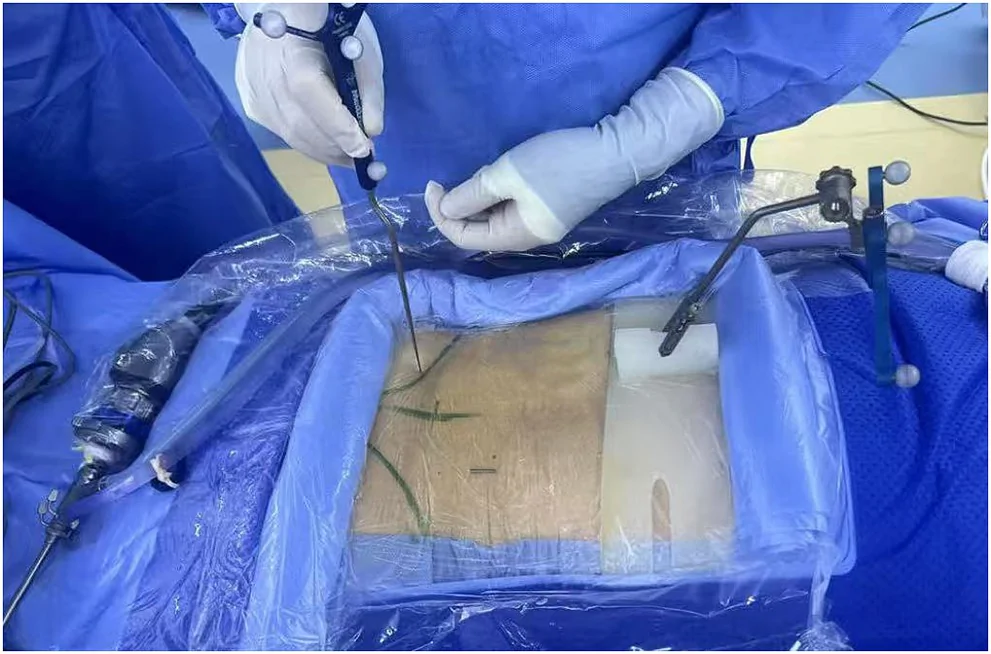
Surgical incision diagram.

**FIGURE 3:**
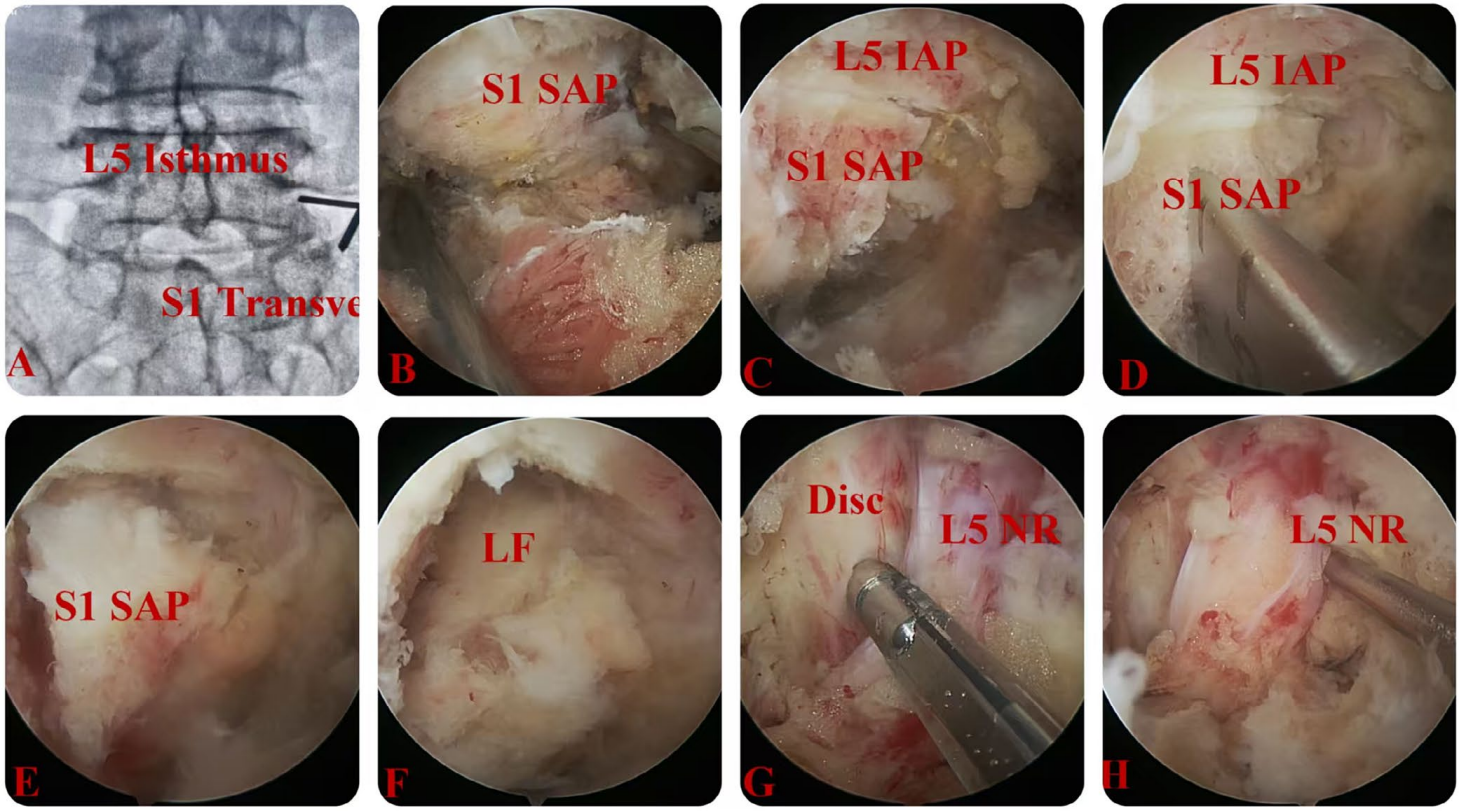
Schematic diagram of UNSES via FLA.

All surgeries were performed by the same surgeon, and the surgical team remained unchanged throughout the study period. The surgical procedure was performed in accordance with our previous published research “Application of the Far‐Lateral Approach in Uni‐portal Non‐coaxial Spinal Endoscopic Surgery: A Delphi Consensus Survey of Expert Opinions,” (1) The middle area between the transverse process of L5 and the superior articular process of S1 is the “landing point”; (2) Remove the lumbosacral ligament to expose the superior articular process of S1 and identify the facet joint; (3) Use a bone knife to remove the tip of the superior articular process, usually 1/3 to 1/2 is appropriate; (4) Remove the superior articular process tip in one piece, and slightly grind the isthmic bone to identify the “true” attachment point of the ligamentum flavum located at the lower edge of the transverse process of L5 and the proximal end of the S1 pedicle; (5) Use forceps to remove the ligamentum flavum, and decide whether to perform the “Turn‐Down” technique based on the situation; (6) Carefully expose the exiting nerve root and intervertebral disc on the ventral side of the fat layer; (7) According to the source of compression for the patient, make individualized choices for the appropriate bony, ligamentous and intervertebral disc decompression range until the nerve root is unobstructed and free from compression [11]. All patients wore a waist support for 6–8 weeks. Patients could get out of bed 24 h after surgery. Anti‐inflammatory and analgesic drugs were given postoperatively to relieve pain, and functional exercise of the lumbar muscles was guided 4 weeks after surgery (Figures 2 and 3).

### 2.3 Grouping Method

The 40 patients were divided into two groups (early stage and proficient stage) based on the principle of consecutive cases without selection bias, so as to truly reflect the learning process of the surgeon. Taking postoperative ODI as the endpoint, with *α* = 0.05, two‐tailed test, observed between‐group mean difference = 4.2, SD = 11.3, total *N* = 40, the calculated statistical power was only 0.31, far below the standard threshold of 0.8.

### 2.4 Outcome Measures

#### 2.4.1 Primary Outcome Measures

(1) Operative time: From the start of incision to the completion of skin suture, excluding the time of anesthesia induction and positioning; (2) Intraoperative blood loss: Measured by the volume of blood in the suction device and the weight of the gauze used during the operation; (3) Fluoroscopy times: The total number of C‐arm fluoroscopies during the operation. Each single C‐arm snapshot exposure counts as one fluoroscopy time; we excluded continuous real‐time screening from counting to match international endoscopic spine research standards. All fluoroscopy data were recounted from surgical video records to guarantee accuracy; (4) complications: nerve injury, cerebrospinal fluid leakage, incision infection, transient lower extremity numbness, and so forth.

#### 2.4.2 Secondary Outcome Measures

(1) VAS score: Used to evaluate the severity of low back pain and lower extremity radiating pain, with a score range of 0–10 points (0 points = no pain, 10 points = severe pain), recorded before surgery and at 1, 3, and 6 months postoperatively. (2) ODI: Used to evaluate the degree of functional disability of patients, with a score range of 0–100 points (0 points = no disability, 100 points = complete disability), recorded before surgery and at 1, 3, and 6 months postoperatively; (3) Modified Macnab criteria: Used to evaluate the clinical efficacy at 6 months postoperatively, including excellent, good, fair, and poor. The excellent and good rate = (number of excellent cases + number of good cases)/total number of cases × 100%.

### 2.5 Learning Curve Fitting

The operative time was taken as the main indicator to fit the learning curve. The scatter plot of the number of surgical cases (*x*‐axis) and operative time (*y*‐axis) was drawn, and the learning curve was fitted by linear regression and CUSUM analysis. The CUSUM analysis was used to determine the inflection point of the learning curve: the cumulative sum of the differences between the actual operative time and the average operative time of all cases was calculated, and the inflection point was the case number where the CUSUM value changed from increasing to stable, which was considered as the point where the surgeon reached proficiency [18, 19, 20].

### 2.6 Statistical Analysis

All data were analyzed using SPSS 26.0 statistical software (IBM, USA). Normally distributed data with homogeneous variance were presented as mean ± standard deviation (*x* ± *s*) and analyzed using one‐way analysis of variance (ANOVA). Comparison of means for continuous variables between groups was performed using a Student *T*‐test, and a Mann–Whitney *U*‐test was used to compare means for nonparametric variables and nonnormal distributions. Categorical data was compared using Pearson *χ* ^2^ and Fisher exact tests as appropriate. Multivariate linear regression analysis was used to explore the factors influencing the operative time. *p* < 0.05 was considered statistically significant.

## 3 Result

### 3.1 General Clinical Data of Patients

A total of 40 patients were included in this study, including 22 males and 18 females, with an age range of 35–68. The surgical levels were L3‐4 (3 cases), L4‐5 (12 cases), and L5‐S1 (25 cases). The disease types included far‐lateral lumbar disc herniation (8 cases), foraminal stenosis (20 cases), and Bertolotti's syndrome (12 cases) (Table 1).

**TABLE 1:** General clinical data of patients.

Item	*N*
Gender (M/F, *n*)	22/18
Age (years, *x̄* ± *s*)	61.8 ± 9.2
Surgical level (*n*)	
L3‐4	3
L4‐5	12
L5‐S1	25
Disease type (*n*)	
Far‐lateral LDH	8
Foraminal stenosis	20
Bertolotti's syndrome	12

### 3.2 Learning Curve Fitting Results

The scatter plot of the number of surgical cases and operative time showed that the operative time gradually decreased with the increase of surgical cases, and the decreasing trend slowed down after a certain number of cases. The linear regression analysis showed that the operative time was negatively correlated with the number of surgical cases (*r* = −0.783, *p* < 0.001). The CUSUM formula = 0.0212*X* ^3^ − 2.0092*X* ^2^ + 47.0051^X^ − 32.1854, *R* ^2^ = 0.9677.

**FIGURE 4:**
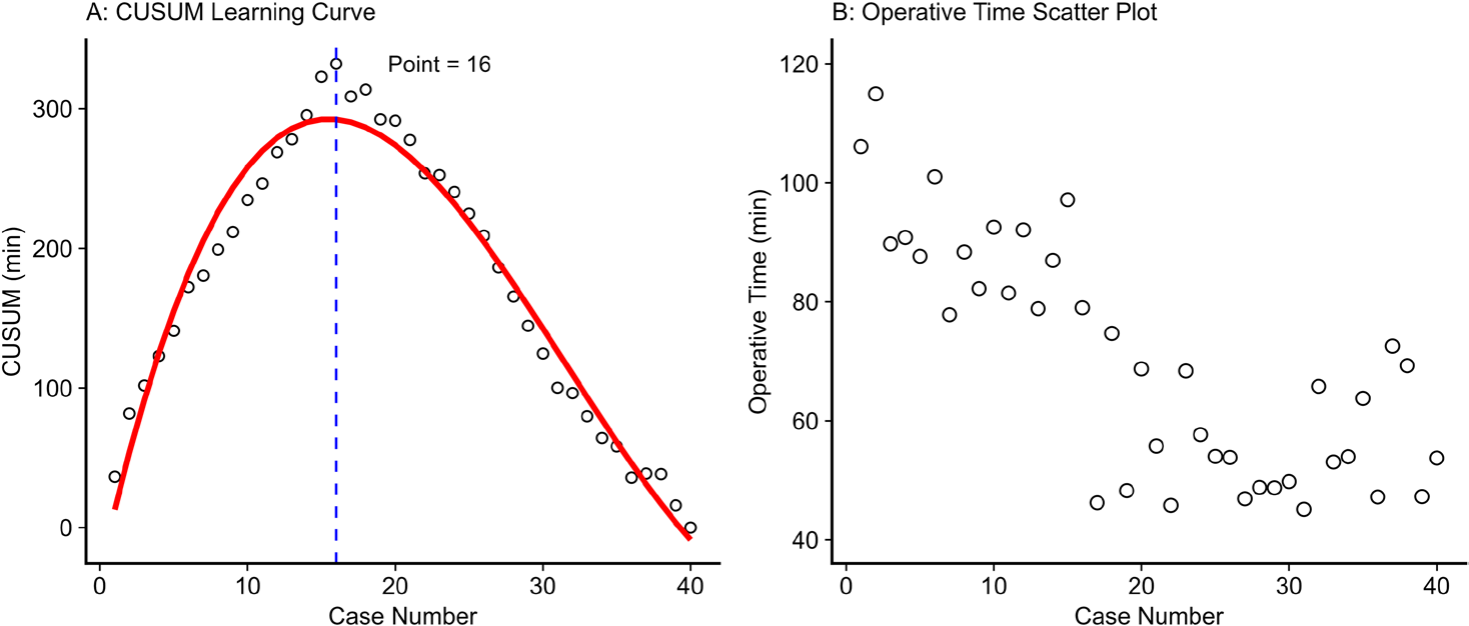
Learning curve of UNSES via FLA.

The CUSUM analysis showed that the cumulative sum of operative time increased continuously in the first 16 cases and began to stabilize after the 16th case, indicating that the inflection point of the learning curve was the 16th case. After the inflection point, the operative time stabilized at (55.3 ± 8.6) min, and the variation range was significantly reduced, indicating that the surgeon had reached the proficient stage (Figure 4).

### 3.3 Primary Outcome Measures

With the increase of surgical cases, the operative time, intraoperative blood loss, and fluoroscopy times showed a significant decreasing trend (*p* < 0.05). The operative time of early stage was (89.5 ± 10.3) min, intraoperative blood loss was (45.2 ± 12.5) mL, fluoroscopy times were (6.6 ± 2.3) times, which were significantly longer or more than that of proficient stage (*p* < 0.05) (Table 2).

**TABLE 2:** Comparison of primary outcome measures between two groups (*x̄* ± *s*).

Domain	Item	Early stage (*n* = 16)	Proficient stage (*n* = 24)
Baseline data	Surgical level (L3‐4, L4‐5, L5‐S1)	1, 4, 11	2, 8, 14
	Disease (LDH, Foraminal stenosis, Bertolotti's syndrome)	3, 8, 5	5, 12, 7
Outcome	Operative time (min)	89.5 ± 10.3	55.3 ± 8.6
	Blood loss (mL)	45.2 ± 12.5	38.1 ± 8.3
	Fluoroscopy times	6.6 ± 2.3	2.4 ± 1.8

The intraoperative complication rate was 18.8% (3/16) in early stage, including 2 cases of dural tear and 1 case of nerve root irritation; 8.3% (2/24) in proficient stage, including 2 cases of dural tear. All complications were successfully managed: the dural tear was repaired with fibrin sealant, and the nerve root irritation was relieved. No serious sequelae such as cerebrospinal fluid leakage or permanent nerve injury occurred.

### 3.4 Secondary Outcome Measures

At 1, 3, and 6 months postoperatively, the VAS score and ODI in two groups were significantly lower than those before surgery (*p* < 0.05). There was no significant difference in postoperative VAS score and ODI between the two groups at each follow‐up time point (*p* > 0.05) (Table 3).

**TABLE 3:** Comparison of VAS score and ODI between two groups (*x̄* ± *s*).

Indicator	Time point	Early stage (*n* = 16)	Proficient stage (*n* = 24)
VAS	Preoperative	7.8 ± 1.2	7.6 ± 1.3
	1 month	2.3 ± 1.0	2.1 ± 0.8
	3 months	2.1 ± 0.8	1.9 ± 0.7
	6 months	1.8 ± 0.7	1.8 ± 0.6
ODI	Preoperative	68.5 ± 10.2	67.8 ± 9.8
	1 month	32.6 ± 8.5	30.8 ± 7.6
	3 months	14.5 ± 7.8	13.6 ± 6.5
	6 months	11.8 ± 6.5	11.5 ± 5.8

According to the modified Macnab criteria at 6 months postoperatively, the excellent and good rate was 87.5% (14/16) in early stage, and 91.7% (22/24) in proficient stage. All the patients achieved satisfactory clinical outcomes, although there was no significant difference between the two groups (*p* > 0.05) (Table 4).

**TABLE 4:** Comparison of Macnab criteria between two groups (*n*, %).

Group	Excellent	Good	Fair	Poor	Excellent/good rate (%)
Early stage (*n* = 16)	7	7	1	1	87.5
Proficient stage (*n* = 24)	10	12	2	0	91.7

### 3.5 Factors Influencing Operative Time

Multivariate linear regression analysis was performed with operative time as the dependent variable, and gender, age, surgical level, disease type, number of surgical cases, and preoperative preparation (whether using imaging software for precise localization) as independent variables. The results showed that the level of surgical cases (L5/SI) (*β* = −0.782, *p* < 0.001) and type of disease (Bertolotti's syndrome) (*β* = −0.156, *p* = 0.028) were independent factors affecting the operative time, indicating that completely mastering the anatomical structure of the complex area could significantly shorten the operative time.

## 4 Discussion

This study enrolled 40 patients, used CUSUM to quantify its learning curve inflection point, and assessed surgical safety and efficacy. The curve inflected at the 16th case; post‐threshold surgery time, fluoroscopy frequency and blood loss dropped sharply, with manageable complications and a satisfying excellent‐good recovery rate. L5/S1 lesions and Bertolotti's syndrome independently prolonged operation time.

### 4.1 Core Learning Curve Characteristics of UNSES‐FLA and Stagewise Skill Evolution

The learning curve in surgery refers to the process in which the surgeon's operational proficiency, surgical efficiency, and clinical outcomes gradually improve with the accumulation of surgical cases. For minimally invasive spinal endoscopic surgery, the learning curve is often steeper than that of traditional open surgery due to the limitations of the surgical field and the need for specialized operational skills, but the specific number of cases varies with the surgical approach and the complexity of the lesion [21, 22, 23].

UNSES via FLA is an innovative minimally invasive spinal surgery technique that has been gradually promoted in recent years, which has unique advantages in the treatment of lumbar degenerative diseases involving the foraminal and extraforaminal regions. However, due to the complex anatomical structure of the lumbar lateral region and the high requirements for endoscopic operation skills, this technique has a certain learning curve, which is an important factor restricting its clinical popularization. This study explored the learning curve of UNSES via FLA by analyzing 40 consecutive cases and found that the inflection point of the learning curve was the 16th case, after which the surgeon's operative proficiency reached a stable level, which provided important evidence for the clinical training of this technique.

Operative time is the most commonly used indicator to evaluate the learning curve of surgical techniques, which can directly reflect the surgeon's operational proficiency and the coordination of the surgical team. In this study, the operative time showed a significant decreasing trend with the increase of surgical cases, from (89.5 ± 10.3) min in the early learning stage to (55.3 ± 8.6) min in the proficient stage, and the CUSUM analysis confirmed that the inflection point was the 16th case. The number of cases in this study was higher than ULBD or lateral recess decompression in our team, which may be related to the complex anatomical structure. The FLA involves the exposure and protection of the transverse process, exit nerve root, and other important structures, and the requirement of foraminoplasty and annulus fibrosus suture increases the difficulty of the operation, so more cases are needed to reach proficiency. In addition to operative time, intraoperative blood loss, fluoroscopy times, and complication rate are also important indicators reflecting the learning curve. In this study, with the accumulation of surgical experience, the intraoperative blood loss and fluoroscopy times gradually decreased, which was due to the improvement of the surgeon's ability to master the anatomical structure and the accuracy of positioning. But the endoscopic blood loss assessment is an indirect estimated value rather than precise direct measurement, and list this as a notable limitation. Of course, we also believe that navigation can significantly reduce the number of surgical fluoroscopy sessions and make the learning curve more gradual.

Before the 16th case, the surgeon was in the initial learning stage and after the 16th case, entered the proficiency stage. This is superior to those research results of other minimally invasive spinal endoscopic technologies, such as PELD or UBE, despite the fact that the surgical strategies employed were different [24, 25, 26, 27, 28]. The main reason for this advantage is that The UNSES technique combines the triangular movements of the arthroscope with the coaxial translation and oscillatory movements of the coaxial spinal endoscope, while expanding upon the unique non‐coaxial rotation technique of UNSES. Investigative and operative tools can be interchanged between the two hands during the procedure, further expanding the operative field and increasing flexibility.

### 4.2 Intraoperative Safety Profiles and Stagewise Variation of Complications

In the initial learning stage, the main problems of the surgeon were: unfamiliar with the anatomical positioning of the FLA, difficult to accurately find the first landing point (isthmus and transverse processes), resulting in more fluoroscopy times; unskilled operation of the endoscope, unclear vision, difficult to effectively remove the compressive substances, resulting in longer operation time and more intraoperative blood loss; insufficient grasp of the scope of bone grinding, easy to cause excessive grinding or insufficient decompression. In the proficiency stage, with the accumulation of clinical experience, the surgeon gradually mastered the anatomical landmarks of the FLA, the positioning accuracy was improved, the fluoroscopy times were reduced, the operation of the endoscopic system was more skilled, the operation time was gradually shortened, and the intraoperative blood loss was reduced. At the same time, the surgeon gradually mastered the scope of bone grinding and nerve root protection skills, and the surgical safety was markedly elevated with fewer nerve irritation events. Furthermore, the surgeon had a proficient grasp of the surgical techniques, could accurately identify the anatomical landmarks, flexibly operate the endoscopic instruments, quickly remove the compressive substances, and effectively protect the nerve root, so the operation time was stable, the fluoroscopy times and intraoperative blood loss were significantly reduced, and the clinical efficacy was significantly improved. We emphasized that surgeons without extensive open spinal experience may require more than 16 cases to reach the inflection threshold; cadaver simulation and supervised proctoring can shorten the learning curve.

The intraoperative complication rate decreased from the early stage to the proficient stage, and the main complications were dural tear and nerve root irritation, which were all successfully managed without serious sequelae. The absolute number of intraoperative adverse events numerically decreased from 3 to 2 cases after the 16th case inflection point, though no statistical difference was detected. This indicates that with the improvement of operational proficiency, the surgeon's ability to avoid and handle complications is enhanced, which is consistent with the research results of spine surgery. The key to reducing complications is to master the anatomical structure of the lumbar lateral region, use intraoperative monitoring, and operate gently.

### 4.3 Clinical Efficacy Analysis

The clinical outcomes of patients are also important manifestations of the learning curve. In this study, there were no significant differences in preoperative VAS score and ODI between the two groups, but the postoperative VAS score and ODI in the proficient stage were slightly lower than those in the early learning stage, and the excellent and good rate of clinical efficacy was significantly higher. Although there was no significant statistical difference in the variations, this indicates that with the improvement of the surgeon's operational proficiency, the decompression effect is more thorough, the damage to the surrounding soft tissue and nerve root is reduced, and the clinical outcomes of patients are significantly improved. In addition, our team's experience also revealed that adequate preoperative preparation (such as precise localization with imaging software) can shorten the operative time, which is consistent with the expert consensus on the application of FLA in UNSES. Preoperative precise localization can help the surgeon quickly find the surgical target, reduce the time of positioning and exploration, and improve surgical efficiency.

The learning curve of UNSES via FLA is affected by many factors, including the surgeon's basic skills, anatomical mastery, preoperative preparation, and team coordination. For surgeons who are new to this technique, we suggest the following measures to shorten the learning curve: First, to strengthen anatomical training, the complex anatomical structure of the lumbar lateral region is the key to the difficulty of the operation. Surgeons should strengthen the learning of lumbar lateral anatomy, and conduct cadaveric anatomical training if it is possible to establish a three‐dimensional anatomical concept. Second, to standardize training, receive systematic training under the guidance of experienced surgeons, learn the key steps and operation skills of the operation, and gradually master the coordination between endoscopic observation and instrument operation. Third, preoperative preparation, use imaging software to conduct precise localization of the surgical level and lesion, formulate a detailed surgical plan, and predict possible complications and treatment measures. Fourth, start with simple cases (such as simple far‐lateral lumbar disc herniation) and gradually transition to complex cases (such as foraminal stenosis and far‐out syndrome) after mastering the basic skills.

### 4.4 Methodological Limitations and Future Research Directions

This study has some limitations. First, it is a single‐center, prospective study with a small sample size; the small underpowered sample may explain all non‐significant inter‐group differences in VAS, ODI, and Macnab excellent‐good rate, and the lack of statistical difference cannot rule out minor clinical improvements. Second, the study only evaluated the learning curve of a single surgeon, and the learning curve may vary among surgeons with different basic skills and experience. Third, the follow‐up time was only 6 months, which only captures short‐term pain/function changes, and the long‐term clinical outcomes and the impact of the learning curve on long‐term recurrence rate need to be further observed. We explicitly stated the 16‐case inflection point only applies to surgeons with 10+ years' surgical background who received limited cadaver pre‐training. In addition, the polynomial regression used to fit the CUSUM curve yields a high *R* ^2^ of 0.9677 yet bears overfitting risk due to the small sample size. This model primarily serves to visually identify the 16th‐case inflection point rather than making predictive inferences for external cohorts. The absence of a control group was also an important limitation of this study. In future, multi‐center, large‐sample studies should be carried out to verify the results of this study, and the learning curve of surgeons with different experience levels should be compared to provide more comprehensive evidence for the clinical promotion and training of UNSES via FLA.

## 5 Conclusions

For selected patients with foraminal/extraforaminal lumbar degenerative lesions, UNSES via FLA achieves favorable short‐term pain relief and functional improvement within the 6‐month follow‐up, with manageable intraoperative complications along with surgeon experience accumulation. The learning curve is relatively steep in the early stage, and the surgeon can reach the proficient stage after 16 cases. With the accumulation of surgical experience, the operation time, blood loss, and fluoroscopy times decreased significantly. The 16‐case proficiency inflection point only applies to surgeons with over 10 years of open spinal surgery background receiving limited cadaver training. Adequate preoperative preparation, proficient mastery of anatomical structures, and standardized training can shorten the learning curve. This study provides a theoretical basis for the clinical promotion and training of UNSES via FLA.

## Author Contributions

**Wenlong Yu:** conceptualization. **Zhichao Ge:** conceptualization. **Jianmin Li:** conceptualization. **Yan Lou:** conceptualization. **Dong Zhang:** conceptualization. **Jiajun Wu:** conceptualization. **Yang Xue:** conceptualization. **Jianfeng Sha:** conceptualization. **Kaipeng Huang:** conceptualization. **Mengchen Yin:** conceptualization. **Shuqiang Wang:** conceptualization. **Hao Sun:** conceptualization. **Kai Lv:** conceptualization.

## Funding

This work was supported by The Key Technology Research Project of “Medical Innovation” Under the Science and Technology Commission of Shanghai Municipality (25Y12800904).

## Consent

The authors have nothing to report.

## Conflicts of Interest

The authors declare no conflicts of interest.

## Data Availability

The data that support the findings of this study are available on request from the corresponding author. The data are not publicly available due to privacy or ethical restrictions.
